# Deep convolutional neural network for weld defect classification in radiographic images

**DOI:** 10.1016/j.heliyon.2024.e30590

**Published:** 2024-05-01

**Authors:** Dayana Palma-Ramírez, Bárbara D. Ross-Veitía, Pablo Font-Ariosa, Alejandro Espinel-Hernández, Angel Sanchez-Roca, Hipólito Carvajal-Fals, José R. Nuñez-Alvarez, Hernan Hernández-Herrera

**Affiliations:** a Postgraduate Program Doctorate in Applied Computer Engineering School of Computer Engineering. University of Valparaiso. Valparaiso, Chile; b Production Engineering Doctorate Postgraduate Program Federal Technological University of Paraná (UTFPR) - Ponta Grossa Campus. PR, Brazil; c Defectoscopy and Welding Technical Services Company, Road O'Burke km. 2½ Pastorita, Cienfuegos, Cuba; d National Center for Applied Electromagnetism (CNEA), Universidad de Oriente, Ave. de Las Américas s/n, 90100, Santiago de Cuba, Cuba; e Intranox SL Pol. La Portalada C/ Circunde, 23 26006, Logroño, La Rioja, Spain; f Pesquisador Visitante. Departamento de Engenharia de Manufatura e Materiais. Universidade Estadual de Campinas. SP, Brazil; g Energy Department, Universidad de la Costa, (CUC), Calle 58 # 55-66, Barranquilla, 080002, Colombia; h Faculty of Engineering, Universidad Simón Bolívar, Carrera 59 #59-132, Barranquilla, 080002, Colombia

**Keywords:** Radiographic testing, Classification, Weld defects, CNNs, Transfer learning

## Abstract

The quality of welds is critical to the safety of structures in construction, so early detection of irregularities is crucial. Advances in machine vision inspection technologies, such as deep learning models, have improved the detection of weld defects. This paper presents a new CNN model based on ResNet50 to classify four types of weld defects in radiographic images: crack, pore, non-penetration, and no defect. Stratified cross-validation, data augmentation, and regularization were used to improve generalization and avoid over-fitting. The model was tested on three datasets, RIAWELC, GDXray, and a private dataset of low image quality, obtaining an accuracy of 98.75 %, 90.255 %, and 75.83 %, respectively. The model proposed in this paper achieves high accuracies on different datasets and constitutes a valuable tool to improve the efficiency and effectiveness of quality control processes in the welding industry. Moreover, experimental tests show that the proposed approach performs well on even low-resolution images.

## 1 Introduction

Welding is widely used in various modern industries, including energy, building construction, ships, automobiles, aircraft, petrochemicals, food, nuclear power, and electronics [[1], [2], [3], [4]]. To ensure the reliability and safety of built structures, it is crucial to have an efficient quality control process, including detecting and classifying defects.

In this regard, industrial radiography is a non-destructive testing (NDT) technique [[5], [6], [7]] that allows the inspection of welds and the determination of the presence of internal faults, as well as leaving a permanent documentary record [8,9]. Certified examiners test welded parts under visual inspection [10,11]. The evaluation criteria may vary depending on the inspector, so the result affects effectiveness and accuracy. At the same time, defect detection and classification become difficult due to contrast changes and noise in radiographic images.

The increase in industrial production brings with it the need to test a large volume of samples in a short period, so the fatigue of the worker and the subjectivity of the evaluations make this process unreliable [12]. Due to this, new advanced computational techniques are necessary to improve the quality and efficiency of the welded joint inspection process [[13], [14], [15], [16], [17]].

Several authors have focused their studies on applying artificial intelligence (AI) techniques for welding inspection from radiographic images and defect classification [18]. In this sense, T. W. Liao et al. [19] proposes using statistical classifiers to extract features from noisy radiographic images of cast aluminum with defects. In contrast, other authors have used fuzzy clustering techniques [20] and fuzzy expert systems for defect classification [21]. Research by K. Carvajal et al. and L. Yang et al. [22,23] proposes pattern recognition using neural networks. However, all these previous methods are based on extracting features chosen by groups of experts.

Otherwise, S. Wang et al. [24] uses deep neural networks (DNN) to classify five weld defects: pore, cracks, lack of fusion, penetration, and slag inclusion, achieving 91.36 % classification accuracy but with a sample number of only 220 images. In a similar study [25], proposed a combination of binary classification and flaw detection on steel surfaces with 98 % accuracy when combining both techniques but with low accuracy for measuring crack defects.

In recent years, the excellent results achieved by convolutional neural networks for image classification have been demonstrated [[26], [27], [28], [29]]. This type of deep learning model has been widely used [30,31] because of its advantages in bringing together feature extraction and classification results in a single structure. A study by Khumaidi et al. [32] classified ten classes in 752 radiographic images, achieving 89 % accuracy using transfer learning techniques in a CNN mode. On the other hand, S. Perri et al. [33] obtained 95.8 % accuracy in classifying four types of weld defects using a dataset of 120 images from a webcam.

A recurring problem in the academic community applying deep learning in non-destructive testing is the need for more public, varied, high volume, and high-quality data collections that allow the training, testing, and validation of the models obtained for defect classification [34]. The GDXray collection is currently available [35], which has in its Welds category only 68 images, so additional work of cropping and manual annotation of the images is needed to train a convolutional neural network (CNN). The WDXI collection [36] has 13,766 images of seven different defect types but is private.

Recently, Totino et al. [37] published a public dataset suitable for weld defect classification containing 24407 radiographic images divided into four defects: crack, pore, lack of penetration, and no fault. This dataset provides the scientific community with a working tool to train neural network models for weld defect classification and identification. In addition, when combined with the transfer learning technique in deep learning models, this dataset can further enhance the model's ability to adapt to different scenarios and improve the accuracy of defect classification.

In various machine learning applications, using transfer learning for feature extraction is an effective technique [[38], [39], [40], [41], [42], [43], [44], [45], [46]]. This technique involves using a pre-trained model to extract meaningful features from the input images, which can improve the model's generalization ability and reduce the risk of over-fitting [47,48].

Transfer learning in deep learning models allows for avoiding overfitting, solving the problem of using small datasets, and extracting the features of the images to be classified [38]. Several pre-trained networks such as VGG16 [49], ResNet50 [45], InceptionResNetV2 [50], DenseNet [51], AlexNet [52], and others have served as feature extractors in classification problems [[53], [54], [55]]. These networks were trained using ImageNet [56], making them suitable for classifying 1000 object classes. Still, they do not consider welding defect classes, so it is necessary to use a set of images to train the model according to the problem to be solved.

In this sense, this paper aims to present a new CNN model based on ResNet50, designed to classify defects in radiographic images. Techniques such as stratified cross-validation, data augmentation, and regularization will be selected to improve model generalization and avoid over-fitting. This approach will represent a valuable tool to enhance the efficiency of quality control procedures in the welding industry, even when confronted with poor-quality images.

## 2 Materials and methods

Defects in welding can significantly impact the strength and integrity of joints. This research addresses four types of defects: crack, pore, lack of penetration, and no defect. These discontinuities are highly prevalent in welds and were chosen for analysis based on the criteria established by experts in Cuban industries.

Crack-type defects are cracks or fractures in the weld or the heat-affected zone. They can be microscopic or visible to the naked eye. Cracks can weaken the structure and propagate under service loads, reducing the strength and service life of the material. Cracks are critical and must be eliminated or rigorously controlled. However, detecting them with non-destructive techniques (NDT) can be challenging due to their size and internal location [[57], [58], [59], [60]].

Pore-type defects refer to small cavities or gas bubbles trapped in the weld. They can be spherical or elongated. Porosity reduces the strength and tightness of the weld. It can affect the integrity of the joint. Although not as critical as cracks, porosity should be minimized. NDT techniques can detect porosity, but their sensitivity varies [61,62].

On the other hand, the non-penetration type defect occurs when the weld does not fully penetrate the base material. There may be a gap between the joined parts. The lack of penetration reduces the strength of the joint and can lead to premature failure. Although there are no apparent defects in the weld on the no-defect samples, NDT inspection is still essential to verify the internal quality [63,64].

### 2.1 Data base

**Fig. 1:**
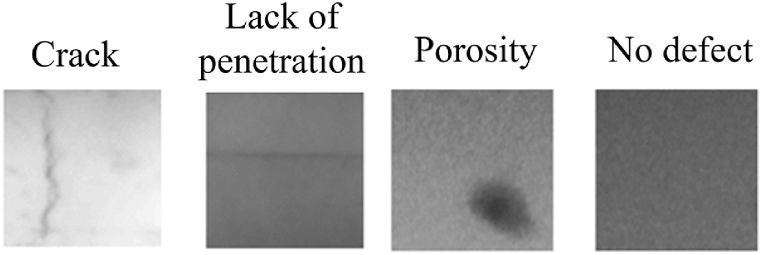
Images of defect types in the RIAWELC dataset [37].

The radiographic image database selected to train the weld defect classification model was RIAWELC [37]. This set has 24,407 radiographic images of size 224 x 224 pixels divided into four types of weld defects: crack, pore, lack of penetration, and no defect, Fig. 1.

For training the neural network, images were selected and balanced, with 1600 samples with 400 images in each class. Class balancing allows for improved model performance, avoids majority class bias, and provides a more accurate evaluation, making it a helpful technique in the classification task [[65], [66], [67]]. The proposed model was trained and validated using a K-fold stratified cross-validation technique [68,69] with K = 5, which allows the dataset to be split into five smaller subsets and used to train and evaluate the model in five rounds. This ensures a more robust and reliable evaluation of the model by using different combinations of training and validation data in each game.

**Fig. 2:**
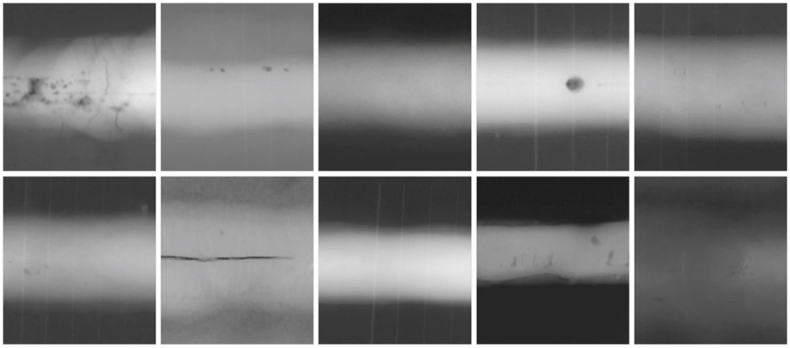
Images of defect types in the GDXray dataset [35].

As a test set, 400 new images from the RIAEWELC database were employed and evenly distributed among the four classes for classification. The generalization assessment of the model was conducted using two image datasets. First, the GDXray dataset [35] was employed. This dataset comprises 68 images in the welding category, necessitating manual cropping and annotation, Fig. 2. The images were resized to 224x224x3 pixels while preserving the three-color channels in PNG format. A total of 100 images were obtained, Table 1.

**Table 1:** Comparison of weld defects in the three data collections: RIAWELC, GDXray, and Private Dataset.

	Weld defect types	Number of images	Sample images	Size of images
**RIAWELC**	Crack Porosity Lack of Penetration No defect	1600		224 x 224
**GDXray**		100		
**Private Dataset**		600		

**Fig. 3:**
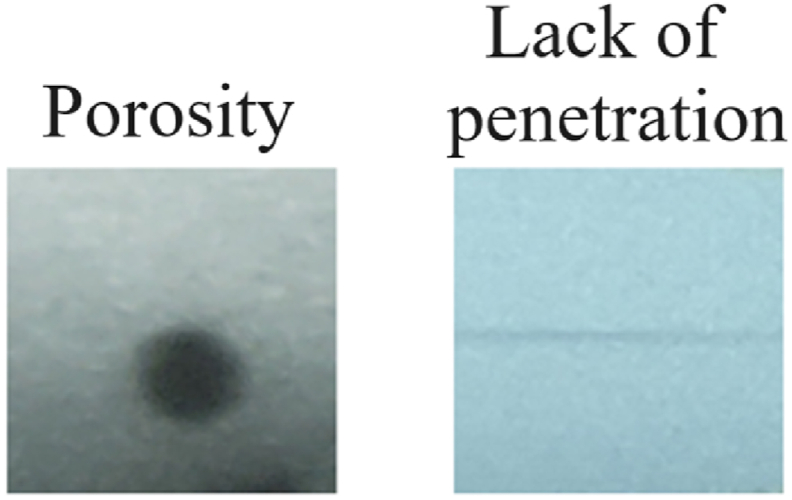
Sample images of porosity defects and lack of penetration in the private image set.

Additionally, a private dataset consisting of radiographic images of welds was utilized. These images constitute the historical archive of the Defectoscopy and Welding Technical Services Company (CENEX) in Cienfuegos, Cuba. Collected over more than 10 years, these images were digitized under non-uniform conditions. Challenges associated with this dataset include low contrast, inconsistent gray distribution, noise, and uneven illumination, Fig. 3.

The images were resized to 224x224x3, retaining all three color channels. To prepare input for the proposed neural network, the region of interest was extracted from the original images, which had dimensions of 9280x6944x3. A total of 600 images were obtained for this dataset, and certified specialists performed labeling from CENEX.

To carry out the experiments, a Google Colab instance with an Nvidia Tesla T4 graphics processor was used to accelerate the processing of the Python code and to take advantage of the Keras and TensorFlow machine learning libraries in the training and evaluation of the model. This platform was used due to its ease of use and availability of computational resources, allowing the experiments to run efficiently and without additional costs. This allowed a higher accuracy of the model and a decrease in training time compared to traditional methods.

### 2.2 ResNet50

ResNet50 is a very effective neural network architecture for image classification and other computer vision problems [28,[70], [71], [72], [73], [74]]. Being pre-trained on large image datasets, ResNet50 can provide high performance without the need to train a neural network from scratch; it is further used to save training time and to aid the effective generalization of the models built by learning from visual patterns and features of the image set it was initially trained on. The use of ResNet50 as a feature extractor in a deep convolutional network for the classification of four types of weld defects in radiographic images is proposed in this paper.

### 2.3 CNN model

**Fig. 4:**
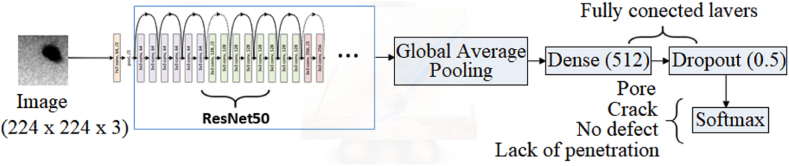
Neural network structure.

In constructing the proposed CNN, taking ResNet50 as the base model, the last classification layer was removed and replaced by four new fully connected layers. The first layer added is a 2D Global Average Pooling layer, which inputs the output of the last convolutional layer of the pre-entered ResNet50 network. Next, a dense layer of 512 neurons with a ReLU activation function is used to reduce dimensionality and learn more abstract and complex features from the images. To avoid overfitting, a Dropout layer was applied at a rate of 50 % after the dense layer. Dropout randomly "deactivates" neurons during training, which helps to regularize the model and reduce dependency on specific features, Fig. 4.

Finally, a last dense layer was added with four neurons, corresponding to the four classes of weld defects to be classified. This layer uses a softmax activation function, which assigns probabilities to each class and allows the classification to be performed.

**Fig. 5:**
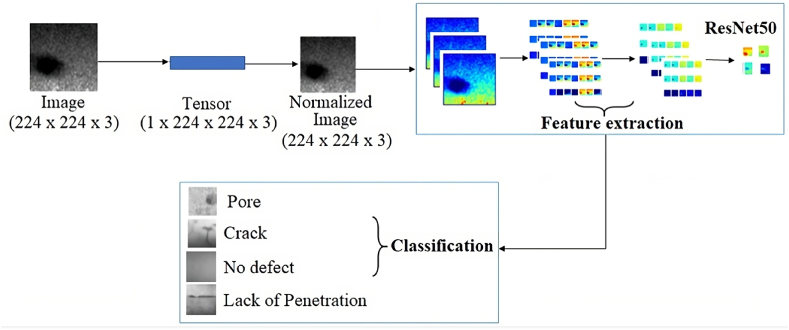
Pre-processing of input images to the CNN network, feature extraction, and classification.

In addition to the architectural modifications mentioned above, the input images were subjected to pre-processing before being fed to the modified CNN. Image pre-processing was performed to ensure compatibility with the ResNet50 base model and improve the input data quality. Image pre-processing involves several steps, Fig. 5. First, the images were converted into tensors, a numerical representation that the neural network can process. The images were in their original format of 224x224 pixels with three color channels (red, green, and blue), so no further size adjustment was necessary.

Normalization was then applied to the image tensors. This involved dividing each tensor value by the standard deviation of the values in the dataset. This normalization helped to scale the pixel values and ensured that the features learned by the model were more stable and consistent during training. Once the images were converted into tensors and normalized, further pre-processing steps were performed, such as data augmentation, which consisted of applying random transformations such as rotation, resizing, cropping, zooming, and horizontal flipping. These transformations generated additional versions of the images during training to improve the model's generalizability and reduce the risk of overfitting. Thus, the initial dataset of 1600 images was expanded to 8000 images.

The modified CNN could extract relevant features and learn from the pre-processed data during training by pre-processing the images in this way, Fig. 5. These pre-processing steps ensured that the images were in a suitable format, with the dimensions 224x224x3, and contained information relevant to the classification task of weld defect detection classification in radiographic images.

Once the classification layers were modified, the previous layers were frozen to avoid changing the general features already learned by the network. This way, a base model was obtained with 1051140 trainable parameters out of 24638852. These layers were then re-trained and adapted to the specific task using the images selected from the RIAWELC dataset. This allowed the network features to be matched to the study of classifying the weld defects in the radiographic images. Table 2 reflects the hyperparameters fitted for this model.

**Table 2:** Hyperparameters used in the training phase of the model.

Parameters	Values
Batch Size	32
Optimizer	Adam
Epochs	50
Learning rate	0.01
Loss function	Categorical Cross-entropy
Data Augmentation	Yes
Number of Classes	4

For fine-tuning, the Adam optimizer was used with a learning rate of 0.01 in 50 training epochs with a batch size of 32 and with the epoch steps calculated as a function of the number of images to adjust the number of iterations required to train the model. Also, a dropout rate of 0.5 was used, a regularization technique that randomly deactivates some of the neurons during training to avoid overdependence on specific features in the training data [75,76]. After fitting the adapted layers, we trained the whole model, including the previously frozen layers, with a low learning rate of 0.0001. This way, a model with improved capability for classifying weld defects in radiographic images was achieved using transfer learning for feature extraction and fine-tuning for task-specific adaptation.

### 2.4 Evaluation of the model

Metrics commonly used to evaluate a neural network model are.

1. Accuracy: As shown in expression (1), it represents the proportion of correctly classified samples.

(1) $$Accuracy=\frac{\left(TP+TN\right)}{\left(TP+TN+FP+FN\right)}$$

2. Recall: Represented in expression (2), it indicates the proportion of positive samples the model correctly identifies.

(2) $$Recall=\frac{TP}{\left(TP+FN\right)}$$

3. Precision: Expressed in expression (3), it signifies the proportion of samples classified as positive that are genuinely positive.

(3) $$Precision=\frac{TP}{\left(TP+FP\right)}$$

4. *F1-Score*: Expressed as expression (4), it combines precision and recall into a single measure.

(4) $$F1-Score=\frac{2\left(\mathrm{Pr}\;ecision*Recall\right)}{\left(Precision+Recall\right)}$$

Where, *TP* = True Positive; *TN* = True Negative; *FP* = False Positive and *FN* = False Negative.

## 3 Results and discussion

Table 3 presents the results obtained during the training, validation, and testing phase on the RIAWELC image dataset. This table compiles the metrics and values obtained at each evaluation stage, providing an overview of the model's performance in accuracy, loss, and other relevant metrics. The results in this table reflect the model's performance in classifying images within the RIAWELC dataset and serve as a benchmark for evaluating the model's effectiveness on the specific task.

**Table 3:** Values of the metrics obtained in the model's training, validation, and testing on the RIAWELC dataset.

Metrics	Values
Train Accuracy (1)	98.60 %
Train Loss	0.145
Validation Accuracy (1)	98.13 %
Validation Loss	0.169
Test Accuracy (1)	**98.75 %**
Test Loss	0.1397
Test Precision (3)	98.75 %
Test Recall (2)	98.75 %
F1-Score Test (4)	99.13 %

The achieved accuracy in the testing phase, with a value of 98.75 %, expression (1), demonstrates the model's ability to perform precise classification on the evaluated dataset. The precision and sensitivity values obtained in the test set, also at 98.5 %, expressions (2) and (3), indicate that the neural network correctly identifies true positives, i.e., welding defects.

The *F1-Score* is a measure that combines precision and sensitivity, providing an overall model performance measure. In this research work, the obtained *F1-Score* was 99.13 %, expression (4), indicating a balance between the accuracy and sensitivity of the model and surpassing those reported in previous research. This is crucial to ensure the reliability and effectiveness of the model in defect detection and classification.

Although there are differences in architectures, amount of training data, and validation, it is essential to note that the results of this research exceed those reported by Totino et al. [37], who used the same RIAWELC dataset and obtained 93.3 % accuracy. The superior performance of the proposed model can be attributed to several factors. First, the structure selected to build the model has proven effective in weld defect classification. Additionally, the model's performance was improved by optimizing the hyperparameters.

**Fig. 6:**
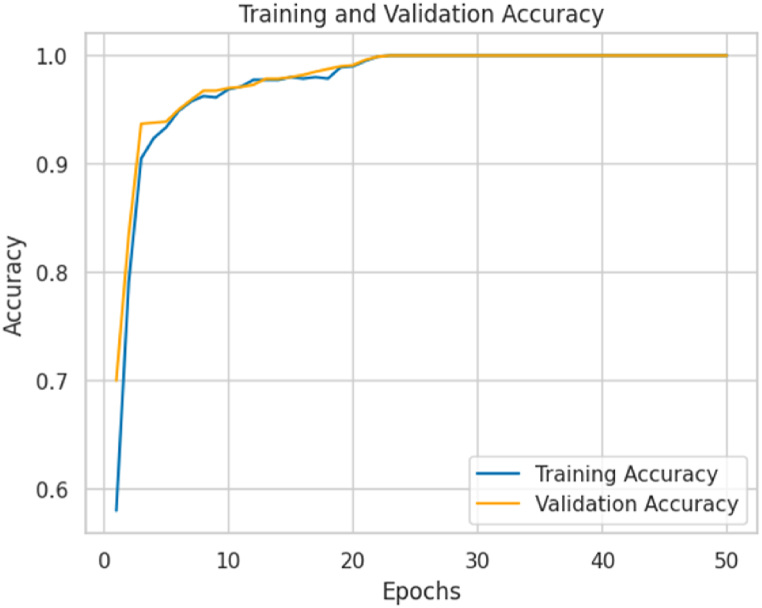
Accuracy curves during training and validation of the proposed model.

Fig. 6 shows the accuracy curves for the training and validation sets. The training curve reaches an accuracy of 98.60 %, while the validation curve reaches 98.13 % at epoch 23, where they begin to converge. These values indicate that the model fits the data well and has learned enough from it. Furthermore, it is observed that accuracy improves rapidly during the first epochs, suggesting effective learning of the model.

This rapid initial improvement indicates the model's generalizability and ability to learn representative patterns. It is important to note that the difference between the accuracy values of the training and validation sets is minimal, which can be attributed to loss during training. The similarity in accuracy between the two sets also supports the model's reliability in classifying weld defects.

The observed consistency in the model metrics, where both training and validation accuracy converge to a similar value, suggests that the model is not experiencing overfitting. Overfitting occurs when a model fits the training data too well and needs help generalizing to new data. When a model shows a significant gap between training and validation accuracy, it indicates overfitting. This means that the model has memorized the training data instead of learning general patterns and features that can be applied to new data. However, in this case, by observing a close convergence between the training and validation metrics, it is inferred that the model has learned effectively and has managed to generalize the knowledge acquired during training to previously unseen data.

**Fig. 7:**
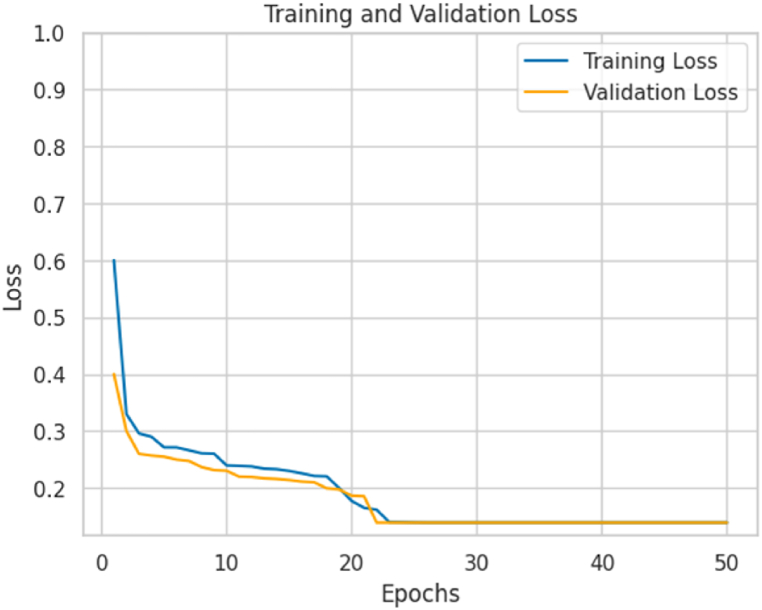
Loss behavior curves during training and validation of the proposed model.

Fig. 7 shows the model's loss behavior in the training and validation phases. A gradual decrease of the loss values is observed for both sets. The loss reached during training is 0.145, while in the validation set, it is 0.169. The curves converge at epoch 23 and do not show a significant decrease after that, showing a similar behavior as in Fig. 6. As the number of training epochs increases, the loss approaches 0, indicating that the model effectively learns and reduces the discrepancy between predictions and accurate labels.

**Fig. 8:**
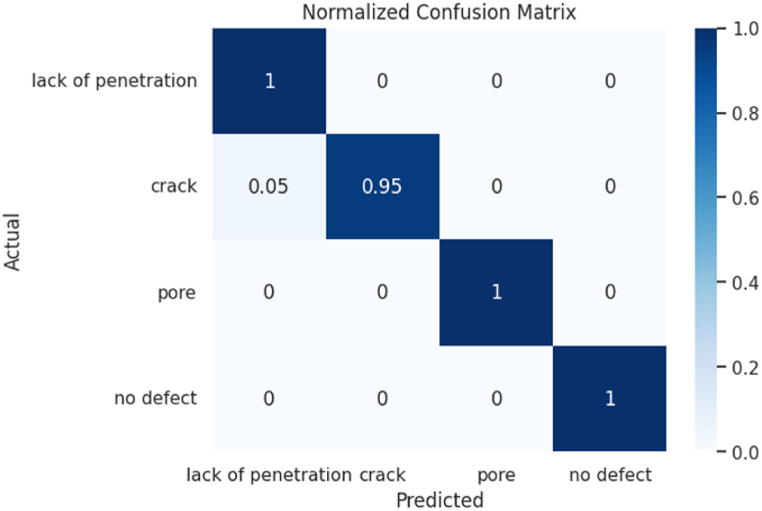
Normalized confusion matrix for the evaluation of the model in the RIAWELC set in the test phase.

The normalized confusion matrix is used to evaluate the performance of a classification model. This matrix represents the relationship between the actual classes and the classes predicted by the model. The normalized confusion matrix shows the frequency of correct and incorrect model predictions for each class, normalized by the total number of instances in that class. For the analysis of the model's performance on the RIAWELC test set, the normalized confusion matrix is shown in Fig. 8. The main diagonal of the normalized confusion matrix indicates the proportion of correctly classified images for each class. In this case, it can be observed that three classes (crack, pore, and no defect) have a value of 1.0 on the main diagonal, which means that the model correctly classified 100 % of these images. However, it is noted that a small percentage of images from the crack class were incorrectly classified (5 % error) as lack of penetration, which may indicate a weakness of the model in recognizing specific patterns or features of that class.

From a mechanical point of view, classifying cracks as non-penetration may be less critical if the cracks in question are superficial and do not compromise structural integrity in strength or durability. In cases where the cracks do not penetrate deeply into the material and do not affect its ability to withstand significant loads, the impact of the classification error may be less. However, it is essential to note that accurate assessment depends on the specific nature of the cracks and the loading conditions applied to the structure.

However, evaluating the model's performance on different datasets is crucial to determine its ability to accurately generalize and classify radiographic images of welds that were not seen during training. Table 4 presents the results of using this CNN model on different test sets, including RIAWELC, GDXray, and our private dataset. Analyzing such samples by the model enables the verification of its ability to adapt to images with diverse characteristics and assesses its generalizability. This additional evaluation of diverse datasets will contribute to a deeper understanding of the model's performance under realistic conditions and strengthen its practical utility in defect classification within radiographic images of welds.

**Table 4:** Model performance on each test data set.

	GDXray	RIAWELC	Private Dataset
Number of images	100	400	600
Size of images	224 x 224	224 x 224	224 x 224
Test Accuracy	90.25 %	**98.75 %**	75.83 %

This analysis facilitates the evaluation of the model's performance in classifying the four types of weld defects across various datasets. From the series of RIAWELC radiographic images, 400 new images not used for training or validation were chosen; an accuracy of 98.75 % was achieved. The GDXray set requires manual cropping and annotation work, and from these, 100 evenly balanced images were selected, achieving an accuracy of 90.25 %. Finally, 600 radiographic images with low quality, low contrast, and uneven illumination were used. These images were divided into equal parts, and an accuracy of 75.83 % was obtained, which validates its use in industrial environments where there is no practical technique for digitizing radiographic plates. The proposed model demonstrates its use for data sets with high-quality and high-density images and other datasets where images are not uniformly digitized.

In the testing phase, the proposed model reaches the lowest accuracy on its dataset, where the quality of the images is poor, but obtains an accuracy of 75.83 %, which can be improved with other pre-processing techniques. Class balancing eliminated the majority class bias so the model could be classified well. The highest accuracy value was obtained when evaluating the model performance on the RIAWELC set with 98.75 %. Assessing the different datasets allowed us to ensure the model's generalization and balance to a specific set of images. The results on the RIAWELC database show an improvement concerning those obtained by D. Mery et al. [34] on the same dataset using the SqueezNet network, getting a 93.3 % test accuracy. However, these authors use more data, which may influence the model's performance.

## 4 Conclusions

This paper proposed a new CNN model based on the ResNet50 architecture to classify four types of weld defects in radiographic images (pore, crack, lack of penetration, and no defect). Regularization-stratified cross-validation and data augmentation techniques were applied to prevent network overfitting and increase generalizability. Performance was tested on the GDXray test set with a test accuracy of 90.25 %, on the RIAWELC set with 98.75 %, and on a private dataset with low-quality images with 75.83 %. The model demonstrated its generalizability by adapting to different image sets. It was evidenced that the proposed neural network, when trained with high-density images in the RIAWELC dataset, can be used to classify welding defects in different types of radiographic image quality of welded parts. It is possible to use this network to classify weld defects in noisy and low-contrast image sets.

Given the results, future adjustments to the model are suggested to improve classification and increase overall accuracy. This may involve refining the algorithms, expanding the training data, or exploring alternative approaches to address pattern detection in the "crack" class and improve classification in noisy, low-contrast images. Therefore, future model tuning is suggested for better classification and greater accuracy. The image dataset should be increased in quantity and variability to optimize the tuning of the neural network hyperparameters and achieve more accurate results, particularly for classifying low-contrast and noisy images.

## Funding

None.

## Data availability statement

The authors confirm that no extra supporting data are available for this article.

No additional information is available for this paper.

## CRediT authorship contribution statement

**Dayana Palma-Ramírez:** Validation, Supervision, Project administration, Methodology, Investigation, Conceptualization. **Bárbara D. Ross-Veitía:** Investigation, Funding acquisition, Formal analysis, Data curation. **Pablo Font-Ariosa:** Validation, Supervision, Resources, Project administration, Methodology. **Alejandro Espinel-Hernández:** Writing – original draft, Visualization, Validation, Supervision, Software, Resources, Investigation, Data curation, Conceptualization. **Angel Sanchez-Roca:** Supervision, Software, Resources, Funding acquisition, Formal analysis, Data curation, Conceptualization. **Hipólito Carvajal-Fals:** Supervision, Resources, Project administration, Investigation, Funding acquisition, Formal analysis. **José R. Nuñez-Alvarez:** Writing – review & editing, Writing – original draft, Visualization, Validation, Formal analysis, Conceptualization. **Hernan Hernández-Herrera:** Writing – review & editing, Writing – original draft, Visualization, Validation, Methodology.

## Declaration of competing interest

The authors declare that they have no known competing financial interests or personal relationships that could have appeared to influence the work reported in this paper.

## References

[1] McPheron T.J., Stwalley R.M. (2022).

[2] Pérez de la Parte M. (2022). Effect of zinc coating on delay nugget formation in dissimilar DP600-AISI304 welded joints obtained by the resistance spot welding process. Int. J. Adv. Des. Manuf. Technol..

[3] Varshney D., Kumar K. (2021). Application and use of different aluminium alloys with respect to workability, strength and welding parameter optimization. Ain Shams Eng. J..

[4] Wang Q. (2021). A tutorial on deep learning-based data analytics in manufacturing through a welding case study. J. Manuf. Process..

[5] Deepak J. (2021). Non-destructive testing (NDT) techniques for low carbon steel welded joints: a review and experimental study. Mater. Today: Proc..

[6] Dwivedi S.K., Vishwakarma M., Soni A. (2018). Advances and researches on non destructive testing: a review. Mater. Today: Proc..

[7] Shaloo M. (2022). A review of non-destructive testing (NDT) techniques for defect detection: application to fusion welding and future Wire arc additive manufacturing processes. Materials.

[8] Eckel S. (2020). Radiographic film system classification and noise characterisation by a camera-based digitisation procedure. NDT E Int..

[9] Szusta J. (2023). Effect of welding process parameters on the strength of dissimilar joints of S355 and Strenx 700 steels used in the Manufacture of Agricultural Machinery. Materials.

[10] Hou W. (2020). Review on computer aided weld defect detection from radiography images. Appl. Sci..

[11] Yahaghi E., Mirzapour M., Movafeghi A. (2021). Comparison of traditional and adaptive multi-scale products thresholding for enhancing the radiographs of welded object. Eur. Phys. J. Plus.

[12] Prunella M. (2023). Deep learning for automatic vision-based recognition of industrial surface defects: a survey. IEEE Access.

[13] Dai W. (2021). Deep learning assisted vision inspection of resistance spot welds. J. Manuf. Process..

[14] Ferguson M.K. (2018). Detection and segmentation of manufacturing defects with convolutional neural networks and transfer learning. Smart and sustainable manufacturing systems.

[15] Han Y., Fan J., Yang X. (2020). A structured light vision sensor for on-line weld bead measurement and weld quality inspection. Int. J. Adv. Des. Manuf. Technol..

[16] Wang D. (2023). Deep network-assisted quality inspection of laser welding on power Battery. Sensors.

[17] Ramírez D.P. (2023). Pore segmentation in industrial radiographic images using adaptive thresholding and Morphological analysis. Trends in Agricultural and Environmental Sciences.

[18] Hermosilla D.M. (2021). Shallow convolutional network excel for classifying motor imagery EEG in BCI applications. IEEE Access.

[19] Mery D. (2023). Pattern recognition in the automatic inspection of aluminium castings, Insight-Non-Destructive Testing and Condition Monitoring.

[20] T. W. Liao, D.-M. Li, Y.-M. Li, Detection of welding flaws from radiographic images with fuzzy clustering methods, Fuzzy Set Syst., 108 (2) (199) 145-158. 10.1016/S0165-0114(97)00307-2.

[21] Liao T.W. (2003). Classification of welding flaw types with fuzzy expert systems. Expert Syst. Appl..

[22] da Silva R.R. (2004). Pattern recognition of weld defects detected by radiographic test. NDT E Int..

[23] Carvajal K. (2004). Neural network method for failure detection with skewed class distribution. Insight-Non-Destructive Testing and Condition Monitoring.

[24] Yang L. (2021). Inspection of welding defect based on multi-feature fusion and a convolutional network. J. Nondestr. Eval..

[25] Wang S. (2021). Automatic detection and classification of steel surface defect using deep convolutional neural networks. Metals.

[26] Say D. (2023). Automated categorization of multiclass welding defects using the x-ray image augmentation and convolutional neural network. Sensors.

[27] Veitía Ross (2020).

[28] Zhang R. (2023). Research on an ultrasonic detection method for weld defects based on neural network architecture search. Measurement.

[29] Li Z., Liu F., Yang W., Peng S., Zhou J. (2022). A survey of convolutional neural networks: analysis, applications, and Prospects. IEEE Transact. Neural Networks Learn. Syst..

[30] Lee K. (2021). Review on the recent welding research with application of CNN-based deep learning part II: model evaluation and visualizations. Journal of Welding and Joining.

[31] Patil R.V., Reddy Y.P. (2023). Multiform weld joint flaws detection and classification by sagacious artificial neural network technique. Int. J. Adv. Des. Manuf. Technol..

[32] Kumaresan S. (2022). Deep learning based Simple CNN weld defects classification using optimization technique. Russ. J. Nondestr. Test..

[33] Singh A., Raj K., Kumar T., Verma S., Roy A.M. (2023). Deep learning-based Cost-effective and Responsive Robot for autism Treatment. Drones.

[34] Perri S. (2023). Welding defects classification through a convolutional neural network. Manufacturing Letters.

[35] Mery D. (2015). The database of X-ray images for nondestructive testing. J. Nondestr. Eval..

[36] Guo W., Qu H., Liang L. (2018). 2018 14th International Conference on Natural Computation, Fuzzy Systems and Knowledge Discovery (ICNC-FSKD).

[37] Totino B., Spagnolo F., Perri S. (2023). RIAWELC: a Novel dataset of radiographic images for automatic weld defects classification. International Journal of Electrical and Computer Engineering Research.

[38] Kumaresan S. (2021). Transfer learning with CNN for classification of weld defect. IEEE Access.

[39] Hernandez-Palma H.G. (2023). Technological tools based on artificial intelligence in the sugar industry: a Bibliometric analysis and future Perspectives for energy efficiency. LADEE.

[40] Kumaresan S. (2023). Deep learning-based weld defect classification using VGG16 transfer learning adaptive fine-tuning. Int. J. Interact. Des. Manuf..

[41] Hussain M., Bird J.J., Faria D.R. (2019). A study on CNN transfer learning for image classification, Advances in Computational Intelligence Systems. Adv. Intell. Syst. Comput..

[42] Jiao W. (2021). End-to-end prediction of weld penetration: a deep learning and transfer learning based method. J. Manuf. Process..

[43] Kumar D.D. (2023). Semi-supervised transfer learning-based automatic weld defect detection and visual inspection. Eng. Struct..

[44] Pan H. (2020). A new image recognition and classification method combining transfer learning algorithm and mobilenet model for welding defects. IEEE Access.

[45] Roy A.M., Bhaduri R. Boseand J. (2022). A fast accurate fine-grain object detection model based on YOLOv4 deep neural network. Neural Comput. Appl..

[46] Roy A.M., Bhaduri J. (2023). DenseSPH-YOLOv5: an automated damage detection model based on DenseNet and Swin-Transformer prediction head-enabled YOLOv5 with attention mechanism. Adv. Eng. Inf..

[47] Rayudu D.V., Roseline J.F. (2023). 2023 International Conference on Artificial Intelligence and Knowledge Discovery in Concurrent Engineering (ICECONF).

[48] Wang X., Yu X. (2023). Understanding the effect of transfer learning on the automatic welding defect detection. NDT E Int..

[49] Nuñez J. (2020). Design of a fuzzy controller for a hybrid generation system. IOP Conf. Ser. Mater. Sci. Eng..

[50] Jiang B., Chen S., Wang B., Luo B. (2022). MGLNN: Semi-supervised learning via Multiple Graph Cooperative learning neural networks. Neural Network..

[51] Li Z., Li Y., Liu Y., Wang P., Lu R., Gooi H.B. (2021). Deep learning based densely connected network for load Forecasting. IEEE Trans. Power Syst..

[52] Singh A., Bruzzone L. (2022). Mono- and Dual-Regulated Contractive-Expansive-Contractive deep convolutional networks for classification of Multispectral Remote sensing images. Geosci. Rem. Sens. Lett. IEEE.

[53] Liu B. (2018).

[54] Mohanasundari L. (2021). Performance analysis of weld image classification using modified Resnet CNN architecture. Turkish Journal of Computer and Mathematics Education (TURCOMAT).

[55] Golodov V.A., Mittseva A.A. (2019). 2019 International Russian Automation Conference (RusAutoCon), Sochi, Russia.

[56] Deng J., Dong W., Socher R., Li L.-J., Li Kai, Fei-Fei Li (2009). 2009 IEEE Conference on Computer Vision and Pattern Recognition.

[57] Chauveau D. (2018). Review of NDT and process monitoring techniques useable to produce high-quality parts by welding or additive manufacturing. Weld. World.

[58] Rao J. (2023). Non-destructive testing of metal-based additively manufactured parts and processes: a review. Virtual Phys. Prototyp..

[59] Priyasudana D. (2023). Double side friction stir welding effect on mechanical properties and corrosion rate of aluminum alloy AA6061. Heliyon.

[60] Ilman M.N. (2021). Microstructure and mechanical properties of friction stir spot welded AA5052-H112 aluminum alloy. Heliyon.

[61] Shin S., Jin C., Rhee J. Yuand S. (2020). Real-time detection of weld defects for automated welding process base on deep neural network. Metals.

[62] Kim D.-Y. (2023). Weld fatigue behavior of gas metal arc welded steel sheets based on porosity and gap size. Int. J. Adv. Des. Manuf. Technol..

[63] Fujii T., Ogasawara N., Tohgo K., Shimamura Y. (2024). Monte Carlo simulation of stress corrosion cracking in welded metal with surface defects and life estimation. Int. J. Mech. Sci..

[64] Li Y. (2019). 2019 IEEE 15th International Conference on Automation Science and Engineering (CASE).

[65] Rai H.M., Chatterjee K. (2022). Hybrid CNN-LSTM deep learning model and ensemble technique for automatic detection of myocardial infarction using big ECG data. Appl. Intell..

[66] Zhu Z., Lin K., Jain A.K., Zhou J. (2023). Transfer learning in deep Reinforcement learning: a survey. IEEE Trans. Pattern Anal. Mach. Intell..

[67] Wang W., Wen G., Zheng Z. (2022). 2022 IEEE 2nd International Conference on Mobile Networks and Wireless Communications (ICMNWC), Tumkur.

[68] Pal K., Patel B.V. (2020). 2020 Fourth International Conference on Computing Methodologies and Communication (ICCMC).

[69] Szeghalmy S., Fazekas A. (2023). A comparative study of the use of stratified cross-validation and distribution-balanced stratified cross-validation in imbalanced learning. Sensors.

[70] Lee J.-R., Ng K.-W., Yoong Y.-J. (2023). Face and facial expressions recognition system for blind people using ResNet50 architecture and CNN. Journal of Informatics and Web Engineering.

[71] Mascarenhas S., Agarwal M. (2021). 2021 International Conference on Disruptive Technologies for Multi-Disciplinary Research and Applications (CENTCON).

[72] Tofigh S., Ahmad M.O., Swamy M.N.S. (2022). A low-Complexity modified ThiNet algorithm for Pruning convolutional neural networks. IEEE Signal Process. Lett..

[73] Huang X. (2023). High resolution Remote sensing image classification based on deep transfer learning and multi feature network. IEEE Access.

[74] Milanés-Hermosilla D. (2021). Monte Carlo dropout for uncertainty estimation and motor imagery classification. Sensors.

[75] Dileep P., Das D., Bora P.K. (2020). 2020 National Conference on Communications (NCC), Kharagpur, India.

[76] Liu Y. (2023). Guided dropout: Improving deep networks without increased computation. Intelligent Automation & Soft Computing.

